# Early increase of CSF sTREM2 in Alzheimer’s disease is associated with tau related-neurodegeneration but not with amyloid-β pathology

**DOI:** 10.1186/s13024-018-0301-5

**Published:** 2019-01-10

**Authors:** Marc Suárez-Calvet, Estrella Morenas-Rodríguez, Gernot Kleinberger, Kai Schlepckow, Miguel Ángel Araque Caballero, Nicolai Franzmeier, Anja Capell, Katrin Fellerer, Brigitte Nuscher, Erden Eren, Johannes Levin, Yuetiva Deming, Laura Piccio, Celeste M. Karch, Carlos Cruchaga, Leslie M. Shaw, John Q. Trojanowski, Michael Weiner, Michael Ewers, Christian Haass, for the Alzheimer’s Disease Neuroimaging Initiative

**Affiliations:** 1 Chair of Metabolic Biochemistry, Biomedical Center (BMC), Faculty of Medicine, Ludwig-Maximilians-Universität München, Munich, Germany; 2 German Center for Neurodegenerative Diseases (DZNE) Munich, Munich, Germany; 3 Department of Neurology, Institut d’Investigacions Biomèdiques, Hospital de la Santa Creu i Sant Pau, Universitat Autònoma de Barcelona, Barcelona, Catalonia Spain; 4 Munich Cluster for Systems Neurology (SyNergy), Munich, Germany; 5 Institute for Stroke and Dementia Research, Klinikum der Universität München, Ludwig-Maximilians-Universität München, Munich, Germany; 6 Izmir International Biomedicine and Genome Institute Dokuz Eylul University, Izmir, Turkey; 7 Department of Neuroscience, Institute of Health Sciences, Dokuz Eylul University, Izmir, Turkey; 8 Department of Neurology, Ludwig-Maximilians-Universität München, Munich, Germany; 9 Department of Psychiatry, Washington University School of Medicine, Saint Louis, MO USA; 10 Department of Neurology, Washington University School of Medicine, St. Louis, MO USA; 11 Hope Center for Neurological Disorders, Washington University in St. Louis, St. Louis, MO USA; 12 Knight Alzheimer’s Disease Research Center, Washington University in St. Louis, St. Louis, MO USA; 13 Department of Pathology and Laboratory Medicine, Perelman School of Medicine, University of Pennsylvania, Philadelphia, PA USA; 14 Center for Neurodegenerative Disease Research, Institute on Aging, Perelman School of Medicine, University of Pennsylvania, Philadelphia, PA USA; 15 University of California at San Francisco, San Francisco, CA USA; 16 Barcelonaβeta Brain Research Center (BBRC), Pasqual Maragall Foundation, Barcelona, Catalonia Spain

**Keywords:** Alzheimer’s disease, Biomarkers, Microglia, Neurodegeneration, Neuroinflammation, Shedding, TREM2

## Abstract

**Background:**

TREM2 is a transmembrane receptor that is predominantly expressed by microglia in the central nervous system. Rare variants in the *TREM2* gene increase the risk for late-onset Alzheimer’s disease (AD). Soluble TREM2 (sTREM2) resulting from shedding of the TREM2 ectodomain can be detected in the cerebrospinal fluid (CSF) and is a surrogate measure of TREM2-mediated microglia function. CSF sTREM2 has been previously reported to increase at different clinical stages of AD, however, alterations in relation to Amyloid β-peptide (Aβ) deposition or additional pathological processes in the amyloid cascade (such as tau pathology or neurodegeneration) remain unclear. In the current cross-sectional study, we employed the biomarker-based classification framework recently proposed by the NIA-AA consensus guidelines, in combination with clinical staging, in order to examine the CSF sTREM2 alterations at early asymptomatic and symptomatic stages of AD.

**Methods:**

A cross-sectional study of 1027 participants of the Alzheimer’s Disease Imaging Initiative (ADNI) cohort, including 43 subjects carrying *TREM2* rare genetic variants, was conducted to measure CSF sTREM2 using a previously validated enzyme-linked immunosorbent assay (ELISA). ADNI participants were classified following the A/T/N framework, which we implemented based on the CSF levels of Aβ_1-42_ (A), phosphorylated tau (T) and total tau as a marker of neurodegeneration (N), at different clinical stages defined by the clinical dementia rating (CDR) score.

**Results:**

CSF sTREM2 differed between *TREM2* variants, whereas the p.R47H variant had higher CSF sTREM2, p.L211P had lower CSF sTREM2 than non-carriers. We found that CSF sTREM2 increased in early symptomatic stages of late-onset AD but, unexpectedly, we observed decreased CSF sTREM2 levels at the earliest asymptomatic phase when only abnormal Aβ pathology (A+) but no tau pathology or neurodegeneration (TN-), is present.

**Conclusions:**

Aβ pathology (A) and tau pathology/neurodegeneration (TN) have differing associations with CSF sTREM2. While tau-related neurodegeneration is associated with an increase in CSF sTREM2, Aβ pathology in the absence of downstream tau-related neurodegeneration is associated with a decrease in CSF sTREM2.

**Electronic supplementary material:**

The online version of this article (10.1186/s13024-018-0301-5) contains supplementary material, which is available to authorized users.

## Background

The triggering receptor expressed on myeloid cells 2 (TREM2) is an innate immune receptor that is expressed on the plasma membrane of microglia in the central nervous system (CNS) [1]. TREM2 is involved in key functions of microglia including phagocytosis, cytokine release, lipid sensing and microglia proliferation and migration [2–6]. *TREM2* mutations strongly increase the risk of developing Alzheimer’s disease (AD) [7, 8] and other neurodegenerative diseases including frontotemporal dementia (FTD), Parkinson’s disease and amyotrophic lateral sclerosis [9–12]. Furthermore, homozygous loss-of-function mutations in *TREM2* are sufficient to cause Nasu-Hakola disease (NHD) and FTD-like syndrome [13, 14]. Together, this suggests that abnormal TREM2 function plays an essential role across different neurodegenerative diseases.

TREM2 is a type-1 transmembrane protein that matures within the secretory pathway and its ectodomain is shed at the plasma membrane [2, 15]. Soluble TREM2 (sTREM2) accumulates in conditioned media of cultured cells and in biological fluids such as plasma and cerebrospinal fluid (CSF) [2, 16]. Shedding is mediated by ADAM10 and 17 C-terminal to histidine 157 [2, 15, 17–19]. Homozygous mutations causing NHD or FTD-like syndrome (such as p.T66M) retain misfolded TREM2 in the endoplasmic reticulum, preventing its maturation and its cleavage on the plasma membrane. Patients bearing these mutations have undetectable levels of sTREM2 in CSF and blood [2, 20, 21].

The fact that TREM2 is selectively expressed in microglia in the CNS and is associated with AD and neurodegeneration, let us hypothesize that sTREM2 in CSF may be a marker for microglia function and its response to Aβ and tau pathology and neurodegeneration. Specifically, sTREM2 may reflect the amount of signaling competent TREM2 on the surface of activated microglia. This idea is supported by the fact that the levels of sTrem2 in the brain of an Aβ mouse model correlate with TSPO small animal positron emission tomography (μPET) signal [22], a marker of microglial activation, and the fact that a *knock-in* mouse model bearing the Trem2 p.T66M mutation has decreased microglial activity [20].

We and others have previously reported changes in the levels of CSF sTREM2 in AD compared to controls [2, 21, 23–26]. Specifically, we found a disease-stage dependent increase in CSF sTREM2 peaking within the early symptomatic stages of late-onset AD [25]. In autosomal dominant AD (ADAD) assessed within the Dominantly Inherited Alzheimer Network (DIAN) project [26], we demonstrated that CSF sTREM2 was increased in mutation carriers compared to non-carriers five years before the estimated years from symptom onset (EYO), but with a considerable delay after the development of Aβ pathology, which emerged about 10-15 years earlier. Together, these studies suggest a complex association of CSF sTREM2 as a function of disease evolution, in which CSF sTREM2 dynamically changes as disease progresses and reaches its highest levels between the later asymptomatic and earlier symptomatic stages, when neurodegeneration has already started.

An important unanswered question in this regard concerns the association between CSF sTREM2 and primary pathologies including Aβ and tau deposition, as well as neurodegeneration during the course of AD. Therefore, we used herein the biomarker-based A/T/N classification system [27], which is the foundation of the recently proposed 2018 NIA-AA research Framework [28]. This classification system consists of three biomarker dimensions including the assessment of Aβ pathology (A), tau pathology (T), and neurodegeneration (N). In the present study, we investigated CSF sTREM2 levels at different AD biomarker-defined groups following the A/T/N classification and the clinical stage (as defined by the clinical dementia rating score, CDR) in participants of the well-characterized ADNI study. This approach allowed us to test the two main aims of this study. First, to assess the association of CSF sTREM2 with Aβ pathology and its downstream pathological processes (i.e. tau pathology and neurodegeneration). Second, to assess the changes on CSF sTREM2 that occur in the Alzheimer’s *continuum* and hence replicate ours and others findings in the ADNI cohort [23–26, 29].

## Methods

### ADNI Participants and study design

This is a cross-sectional study in which CSF sTREM2 was measured in 1031 participants of the ADNI project. Among them, 4 individuals did not have the AD core biomarkers measurements and were further excluded from the analysis, rendering a study sample of 1027 subjects. The CSF sTREM2 measurements were uploaded to the ADNI database (http://adni.loni.usc.edu) on 16/03/2018 and the data used in this study was downloaded on 21/03/2018. The ADNI project (http://www.loni.usc.edu/) is a multicenter longitudinal study led by Principal Investigator Michael W Weiner with the main goal to develop and validate biomarkers for subject selection and as surrogate outcome measures in late-onset AD [30]. The institutional review boards (IRB) of all participating centers approved the procedures of the study and all participants or surrogates provided informed consent. Our local IRB (LMU) also approved the study.

### Clinical classification

In line with the recently published 2018 NIA-AA “research framework” for the diagnosis of Alzheimer’s disease [28], we assigned each ADNI participant in a group defined by its biomarker profile, as described by the A/T/N scheme [27], coupled with its cognitive status, as defined by the CDR score [31]. The A/T/N scheme comprises 3 biomarker groups: “A” refers to aggregated Aβ, “T” aggregated tau and “N” to neurodegeneration. Each biomarker group is binarizied in negative (-) or positive (+) based on whether their biomarkers are normal or abnormal. In the present study, we assigned “A+” to those individuals that had a CSF Aβ_1-42_ < 976.6 pg/ml, “T+” to those individuals with P-tau_181P_ > 21.8 pg/ml and “N+” to those individuals with T-tau > 245 pg/ml. We merged the aggregated tau (T) and neurodegeneration (N) groups in order to decrease the number of groups to be compared. TN negative (TN-) was defined as having both the aggregated tau (T) and neurodegeneration (N) biomarkers in the normal range (T- and N-, that is P-tau_181P_ ≤ 21.8 pg/ml and T-tau ≤ 245 pg/ml). Participants were classified as TN positive (TN+) if either aggregated tau (T) or neurodegeneration (N) were abnormal (T+ or N+, that is P-tau_181P_ > 21.8 pg/ml or T-tau > 245 pg/ml). Only 5.4% of the individuals of the total differed between the T and N biomarkers groups.

The combination of the biomarker profile (A/T/N scheme) and the clinical status (CDR) rendered 12 different groups that are displayed in Table 1. We studied CSF sTREM2 in ADNI following two approaches. In a first one, we compared CSF sTREM2 between the different A/T/N categories within each clinical stage. In a second one, we attempted to model the course of AD with biomarker and clinical-based groups, similar to what was proposed by the 2011 NIA-AA criteria [32–34]. Thus, in this second approach, we compared the ‘CDR = 0 A-/TN-’ group (which corresponds to healthy controls) with those biomarker-based groups that fall into the Alzheimer’s *continuum* category, that is: ‘Preclinical AD A+/TN-’, ‘Preclinical AD A+/TN+’, ‘AD CDR = 0.5’ and ‘AD CDR = 1’. Since our aim was to study the Alzheimer's *continuum*, we excluded from this analysis those individuals that fall in the category of suspected non-Alzheimer’s pathology (SNAP) [35–38] and those symptomatic individuals (CDR > 0) that do not have positive biomarkers for both Aβ deposition (CSF Aβ_1-42_) and neurodegeneration/tau pathology (T-tau or P-tau_181P_).

**Table 1:**
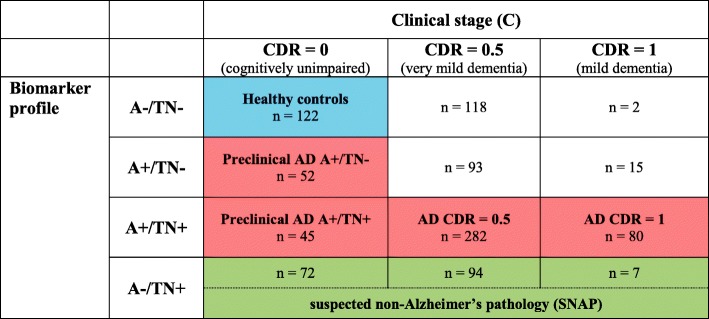
Classification of ADNI participants based on the A/T/N framework and clinical stage

The ADNI participants were classified based on their clinical stage, as defined by the clinical dementia rating (CDR) score, and the biomarker-based A/T/N framework. The A/T/N framework comprises 3 biomarker groups: *A* Aβ pathology biomarker status, *T* tau pathology biomarker status, and *N* neurodegeneration biomarker status. Each of the biomarkers group have binarized into positive/abnormal (+) or negative/normal according the biomarkers cutoffs. T and N have been merged to simplify the classification and TN- indicates that both T and N are normal and TN+ indicates that T and/or N are abnormal.

The numbers shown here are excluding the *TREM2* mutation carriers and CSF sTREM2 outliers (as defined as values 3 standard deviations above or below the mean).

The colour indicates the different groups used for comparisons in the main text. Healthy controls (*n* = 122) are depicted in blue, the Alzheimer’s *continuum* (*n* = 459) in red and the suspected non-Alzheimer’s pathology (SNAP) group (*n* = 173) in green.

Bold text depicts the groups analysed when modelling the course of AD, namely 'healthy controls', 'Preclinical AD A+/TN -', 'Preclinical AD A+/TN+', 'AD CDR = 0.5' and 'AD CDR = 1'

### Alzheimer’s disease CSF core biomarkers and CSF sTREM2 measurements

In the present study, we used the AD CSF core biomarkers measurements performed with the Elecsys® total-tau CSF, the Elecsys® phosphotau(181P) CSF and the Elecsys® β-amyloid(1–42) CSF immunoassays on a cobas e 601 instrument [39, 40]. The data is available in the ‘UPENNBIOMK9.csv’ file in the ADNI database. These immunoassays are for investigational use only. They are currently under development by Roche Diagnostics and not commercially available yet. The analyte measuring ranges (lower technical limit to upper technical limit) of these assays are the following: 80 to 1300 pg/ml for total-Tau CSF, 8 to 120 pg/ml for phosphor-Tau(181P) CSF, and 200 to 1700 pg/ml for Elecsys® β-Amyloid(1-42) CSF immunoassays. The measuring range of the Elecsys® β-Amyloid(1-42) CSF immunoassay beyond the upper technical limit has not been formally established. Therefore use of values above the upper technical limit, which are provided based on an extrapolation of the calibration curve, is restricted to exploratory research purposes and is excluded for clinical decision making or for the derivation of medical decision points.

CSF sTREM2 measurements were done with a MSD platform-based assay, previously reported and validated [2, 25, 26]. A comprehensive description of the assay is shown in Supplementary methods (see Additional file 1: Supplementary methods). The CSF sTREM2 measurements are publicly available in the ADNI database.

### Cell culture and transient transfection of HEK293T cells

HEK293T cells were cultured in DMEM with Glutamax I supplemented with 10 % (v/v) fetal calf serum (FCS) and 1 % penicillin/streptomycin. 24 hours after seeding, cells were transiently transfected with equal amounts of DNA coding for the different *TREM2* variants using Lipofectamine 2000 as the transfection reagent. *TREM2* variant constructs were generated by site-directed mutagenesis (Stratagene) of a *TREM2 wt* construct bearing N- and C-terminal HA and FLAG tags, respectively, as previously described [2]. All constructs were verified by DNA sequencing (GATC Biotech). We collected the conditioned media 48 hours after transfection. Cellular debris was removed by centrifugation at 4°C (13300 rpm, 20 min). Supernatants were subsequently frozen at -20°C until analyses were performed. Cell culture reagents were purchased from Thermo Fisher Scientific unless otherwise noted.

We measured the concentrations of HA-labeled TREM2 protein in the HEK293T conditioned media by two different ELISAs. First, sTREM2 concentrations were determined by the same ELISA used to measured sTREM2 in the human CSF samples, which includes a detection antibody against sTREM2 (see Additional file 1: Supplementary methods). Second, it was measured by a MSD platform-based including an antibody against the HA-tag. This second assay follows the same protocol as the first one but with the following modifications. The detection antibody is a monoclonal rat IgG anti-HA peptide sequence (YPYDVPDYA), clone 3F10 (Roche, Cat. No. 11 867 423 001; 100 ng/mL, 50 μL/well); the secondary antibody is a SULFO-TAG-labeled goat polyclonal anti-rat IgG antibody (MSD, Cat. no. R32AH-1; 0.5 μg/mL, 25 μL/well). The samples were diluted 1:50 and 1:100 in assay buffer [0.25% BSA and 0.05% Tween 20 in PBS (pH = 7.4)], supplemented with protease inhibitors (Sigma; Cat. no. P8340) and measured in duplicates for each dilution. We acquired the electrochemiluminescence response values using the MESO QuickPlex SQ 120. We compared the signal of the sTREM2 ELISA with that of the HA-tag assay for each of the TREM2 rare variants. The percentage between these two assays renders a relative affinity of the sTREM2 ELISA to each of the TREM2 rare variants in relation to its respective HA-tag control.

### Statistical analysis

CSF sTREM2 did not follow a normal distribution (Kolmogorov-Smirnov test: *P <* 0.0001) and were hence log_10_-transformed. After transformation, CSF sTREM2 followed a normal distribution as assessed by Kolmogorov-Smirnov test (*P =* 0.200) and visual inspection of the histogram. All the statistical analysis described in this study are performed with the log_10_-transformed values.

A one-way analysis of covariance (ANCOVA) was conducted to determine statistically significant differences on CSF sTREM2 between *TREM2* rare variants carriers and the non-carriers’ individuals adjusting for the effect of age, followed by a Bonferroni corrected *post hoc* pairwise comparison. Only those groups of *TREM2* rare variants carriers that comprise more than 1 subject were included in the analysis.

The following analyses were conducted excluding outliers’ values of CSF sTREM2, defined as values differing 3 standard deviations from the mean. There were 5 outliers: 2 subjects classified as ‘Preclinical AD A+TN-’ (1 a *TREM2* rare variant carrier and 1 a non-carrier), 1 classified as ‘CDR = 0.5 A+TN-’ (*TREM2* rare variant carrier), 1 classified as ‘AD CDR = 0.5’ (*TREM2* rare variant carrier), 1 classified as ‘AD CDR = 1’ (non-carrier). Including or excluding these outliers do not change the findings of this study.

To study the association of CSF sTREM2 with demographic and genetic data, we computed a linear regression model with CSF sTREM2 as an outcome variable and age, gender and *APOE* ε4 status as fixed effects. Since only age showed to be a significant predictor of CSF sTREM2, the following analyses were conducted including only age as a covariate.

To test the differences in CSF sTREM2 across biomarker profiles in the A/T/N framework, we applied a one-way ANCOVA including age as covariate, followed by Bonferroni corrected *post hoc* pairwise comparisons. A similar approach was used to test whether CSF sTREM2 changes across the Alzheimer’s *continuum*. These analyses were performed including or excluding individuals carrying a *TREM2* rare variant and yielded similar results.

Finally, we studied the association between CSF sTREM2 and each of the CSF core biomarkers for AD (T-tau, P-tau_181P_, Aβ_1-42_) with a multiple linear regression adjusted for age. The analysis was conducted separately in the healthy controls, Alzheimer’s *continuum* and SNAP groups. We performed the analysis both including or excluding outliers (defined as AD CSF core biomarkers 3 standard deviations below or above the group mean) in order to exclude that the associations were driven by extreme values. The analysis with and without outliers rendered similar results. For CSF Aβ_1-42_, the analyses were performed using both the truncated values at the upper technical limit and the exploratory measurements available based on the extrapolation of the calibration curve. In the main text, we report the results using the extrapolated measurements, but using the truncated ones yielded similar results.

Statistical analysis was performed in SPSS IBM, version 20.0, and the free statistical software R (http://www.r-project.org/). Figures were built using GraphPad Prism or free statistical software R. All tests were 2-tailed, with a significance level of α = 0.05.

## Results

### Association of CSF sTREM2 with genetic and demographical data

**Fig. 1:**
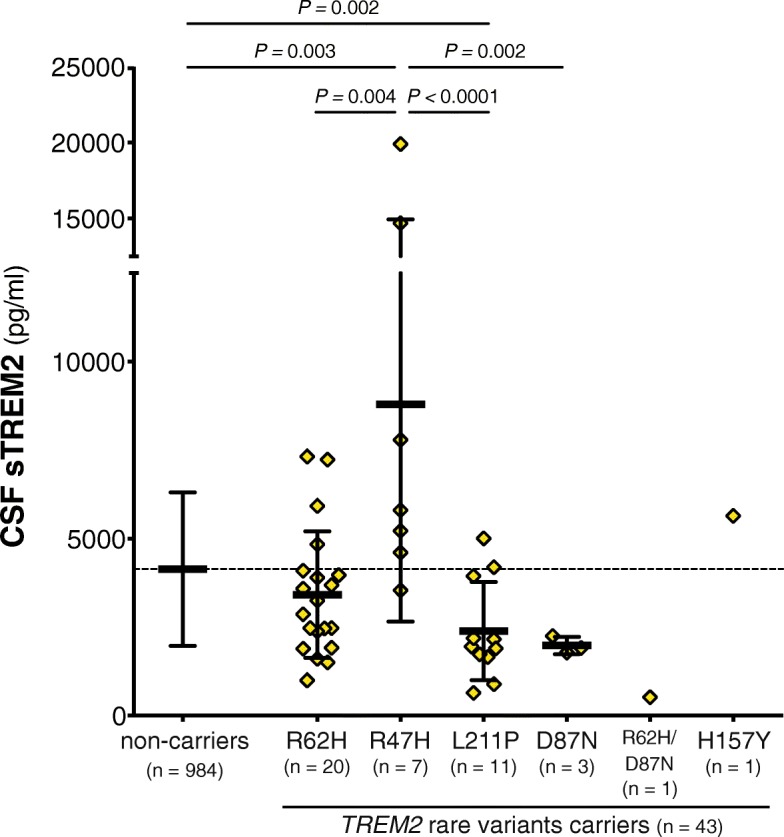
CSF sTREM2 in ADNI participants carrying a *TREM2* rare variant. Scatter plot representing the levels of CSF sTREM2 in carriers of a *TREM2* rare variant, compared to the non-carriers ADNI participants. Solid bars represent the mean and the standard deviation (SD). *P*-values were assessed by a one-way ANCOVA adjusted for age, followed by Bonferroni corrected *post hoc* pairwise comparisons between the *TREM2* variants carriers’ groups and the non-carriers. We did not include in the comparison those rare variants with only one subject (p.R62H/p.D87N and p.H157Y).

We studied a total of 1027 participants of the ADNI study. The demographical and clinical characteristics of the whole study population are described in Table S1 (see Additional file 1). Among the participants studied, 43 (4.2 %) had a known *TREM2* rare variant (see reference [41] for a comprehensive review of the pathogenicity of each variant). The overall mean levels of CSF sTREM2 of these individuals (M = 3913 pg/ml, SD = 3548, n = 43) were significantly lower than the rest of ADNI participants without a *TREM2* rare variant (M = 4136 pg/ml, SD = 2171, n = 984; F_1,1024_ = 6.77, *P* = 0.009, η_p_^2^ = 0.007; Table 2, Fig. 1) in a one-way ANCOVA adjusted for age. However, CSF sTREM2 varied considerably between *TREM2* variants (F_4,1019_ = 8.79, *P* < 0.0001, η_p_^2^ = 0.033) and Bonferroni’s *post hoc* comparisons test indicated that the p.R47H variant [7–12] had significantly higher CSF sTREM2 (*P* = 0.003) and the p.L211P variant [42, 43] significantly lower CSF sTREM2 (*P* = 0.002) than non-carriers. No differences in CSF sTREM2 were found between individuals with a p.R62H [44, 45] and the p.D87N [7] variants and the non-carriers. There was a single subject carrying both a p.D87N and p.R62H variants, and another single subject carrying a p.H157Y variant, which were not included in the statistical analysis. However, it is worth noting that the subject carrying a p.H157Y *TREM2* rare variant had relatively high CSF sTREM2 (Table 2, Fig. 1), an observation that agrees with our previous findings that the p.H157Y variant, which is located exactly at the cleavage site, increases shedding of TREM2 [17]. Given that *TREM2* rare variants may influence CSF sTREM2 (as described here and in [24]), all the following analyses are excluding participants carrying these rare variants. Nevertheless, including the *TREM2* rare variants carriers did not change the results. In order to test whether the differences in CSF sTREM2 among *TREM2* rare variants are influenced by differences in the antibody affinity to the mutant sTREM2, we transfected HEK293T cells with an epitope tagged *wild type* (*wt*) and mutated TREM2 and measured sTREM2 released in the media with the same ELISA used for the quantification of CSF sTREM2 and with an ELISA using an antibody against the epitope tag (Additional file 1: Figure S1). This revealed that the p.R47H, p.R62H and p.H157Y *TREM2* rare variants were detected with a slightly reduced efficiency in our ELISA; therefore, the increased levels of CSF sTREM2 found in subjects bearing the p.R47H rare variants are even slightly underestimated. On the other hand, the p.L211P *TREM2* rare variants were detected efficiently, independently of their individual amino acid exchanges. However, the p.D87N *TREM2* rare variant was detected with significant less affinity in our ELISA than using the antibody against the epitope tag. Thus, the decreased CSF sTREM2 found in the p.D87N rare variant should be interpreted with caution.

**Table 2:** Demographic and clinical characteristics of the individuals carrying a *TREM2* rare variant

	Non-carriers (*n* = 984)	p.R62H (*n* = 20)	p.R47H (*n* = 7)	p.L211P (*n* = 11)	p.D87N (*n* = 3)	p.R62H/D87N (*n* = 1)	p.H157Y (*n* =1)
Age, y	73.1 (7.35)	74.7 (6.47)	73.5 (11.3)	72.8 (4.36)	72.7 (6.17)	66.4	73.1
Female, n (%)	430 (43.7)	11 (55.0)	3 (42.9)	6 (54.5)	0	0	1
*APOE* ε4 carriers, n (%)	467 (47.5)	8 (40.0)	5 (71.4)	2 (18.2)	2 (66.7)	0	0
Education, y	16.0 (2.78)	15.7 (2.39)	15.6 (2.07)	14.6 (2.54)	17.0 (2.65)	15.0	18.0
CSF biomarkers (pg/ml)							
T-tau	289 (136)	322 (140)	353 (125)	231 (119)	299 (100)	116	214
P-tau_181P_	27.9 (14.9)	30.9 (15.6)	36.4 (15.8)	22.2 (12.7)	27.9 (11.5)	9.92	18.1
Aβ_1-42_	982 (457)	1073 (437)	874 (454)	1246 (515)	944 (670)	925	1700
sTREM2	4136 (2171)	3418 (1786)	8790 (6136)	2386 (1390)	1981 (244)	518	5642
Associated diseases	na	AD	AD, FTD, PD, ALS	AD, FTD	AD	AD	AD
References	na	[44, 45]	[7–12]	[42, 43]	[7]	[7, 44, 45]	[56]

Data are expressed as mean and standard deviation (SD) or number (n) and percentage (%), as appropriate.

Abbreviations: *Aβ*_*1-42*_ amyloid-β 42, *AD* Alzheimer’s disease, *ALS* amyotrophic lateral sclerosis, *APOE* apolipoprotein E, *CSF* cerebrospinal fluid, *FTD* frontotemporal dementia, *na* non-applicable, *PD* Parkinson’s disease, *P-tau*_*181P*_ tau phosphorylated at threonine 181, *T-tau* total tau, *y* years.

In the sample excluding the *TREM2* rare variants carriers and TREM2 outliers’ values (n = 982, see methods section), we first assessed which demographic and genetic variables are associated with CSF sTREM2 (descriptives summarized in Table 3). Consistent with previous results [21, 23–26], CSF sTREM2 levels were associated with age (β = +0.275, *P* < 0.0001, η_p_^2^ = 0.073), but not with gender (F_1,978_ = 0.029, *P* = 0.866, η_p_^2^ = 0.00003) or *APOE* ε4 status (F_1,978_ = 0.099, *P* = 0.753, η_p_^2^ = 0.0001). Consequently, all further analysis included age as a covariate, but not gender or *APOE* ε4 status.

**Table 3:** Demographic and clinical characteristics of the sample excluding the individuals carrying a *TREM2* rare variant

	CDR = 0 (n = 291)				CDR = 0.5 (n = 587)				CDR = 1 (n = 104)			
	A-/TN- *(n* = 122)	A+/TN- (*n* = 52)	A+/TN+ (*n* = 45)	A-/TN+ (*n* = 72)	A-/TN- (*n* = 118)	A+/TN- (*n* = 93)	A+/TN+ (*n* = 282)	A-/TN+ (*n* = 94)	A-/TN- (*n* = 2)	A+/TN- (*n* = 15)	A+/TN+ (*n* = 80)	A-/TN+ (*n* = 7)
Age, y	72.5 (5.50)	73.5 (5.95)	76.3 (5.46)	74.6 (6.56)	69.9 (7.56)	72.6 (7.74)	73.3 (7.08)	73.2 (8.16)	89.2 (1.63)	76.3 (6.05)	74.1 (9.28)	79.8 (8.02)
Female, n (%)	58 (47.5)	24 (46.2)	24 (53.3)	40 (55.6)	55 (46.6)	21 (22.6)	119 (42.2)	42 (44.7)	0 (0)	5 (33.3)	40 (50.0)	2 (28.6)
*APOE* ε4 carriers, n (%)	17 (13.9)	20 (38.5)	27 (60.0)	15 (20.8)	24 (20.3)	47 (50.5)	216 (76.6)	32 (34.0)	0 (0)	8 (53.3)	59 (73.8)	1 (14.3)
Education, y	16.4 (2.79)	16.0 (2.65)	16.5 (2.55)	16.4 (2.57)	16.0 (2.68)	16.1 (2.97)	15.9 (2.90)	15.9 (2.66)	17.0 (0)	16.2 (2.51)	15.1 (2.80)	15.0 (2.08)
CSF biomarkers, pg/ml*												
T-tau	185 (32.2)	167 (40.5)	332 (79.3)	322 (71.9)	183.4 (38.2)	173 (40.0)	381 (136)	338 (111)	234 (6.08)	188 (38.8)	393 (136)	489 (221)
P-tau_181P_	16.2 (2.88)	15.4 (4.07)	33.3 (9.09)	28.9 (7.43)	15.8 (3.32)	15.9 (4.14)	38.9 (14.7)	31.6 (13.7)	19.3 (1.20)	17.4 (3.53)	39.2 (15.0)	43.2 (22.9)
Aβ_1-42_	1456 (223)	723 (196)	717 (168)	1538 (235)	1428 (247)	632 (196)	634 (168)	1427 (290)	1330 (14.9)	538 (185)	570 (159)	1484 (290)
sTREM2	3741 (1690)	2835 (1524)	4839 (2240)	5378 (2147)	3436 (1754)	2791 (1292)	4441 (2211)	5478 (2370)	5660 (2008)	3051 (1128)	3967 (2000)	7619 (3539)

Data are expressed as mean and standard deviation (SD) or number (n) and percentage (%), as appropriate.

*The CSF core biomarkers measurements were performed using the electrochemiluminiscence immunoassays Elecsys Total-tau CSF, phosphor-tau(181P) CSF and β-amyloid(1-42) CSF, which have an upper technical limit of 1300 pg/ml (T-tau), 120pg/ml (P-tau181P) or 1700 pg/ml (Aβ_1-42_). The values above these limits were truncated to the respective upper technical limit.

Abbreviations: *A* Aβ pathology biomarker status, *Aβ*_*1-42*_ amyloid-β 42, *APOE* apolipoprotein E, *CDR* clinical dementia rating, *CSF* cerebrospinal fluid, *N* neurodegeneration biomarker status, *P-tau*_*181P*_ tau phosphorylated at threonine 181, *T* tau pathology biomarker status, *T-tau* total tau, *y* year

### Differences of CSF sTREM2 within the A/T/N classification of AD

In order to assess the impact of Aβ deposition or the downstream processes of the amyloid cascade (i.e. tau pathology and neurodegeneration), we applied the recently proposed A/T/N classification framework of AD, which proposes 3 binary biomarker groups [27]: (1) aggregated Aβ (A+/A-), (2) aggregated tau (T+/T-) and (3) neurodegeneration (N+/N-). Given that CSF T-tau and CSF P-tau_181P_ were highly correlated, we merged the tau (T) and neurodegeneration (N) groups. ‘TN-’ profile was defined as both CSF P-tau_181P_ and T-tau within the normal range, whereas ‘TN+’ was defined as abnormal levels of CSF P-tau_181P_ or T-tau. Thus, we compared 4 different biomarker profiles within each clinical stage, namely: (1) A-/TN-, (2) A+/TN-, (3) A+/TN+ and (4) A-/TN+.

**Fig. 2:**
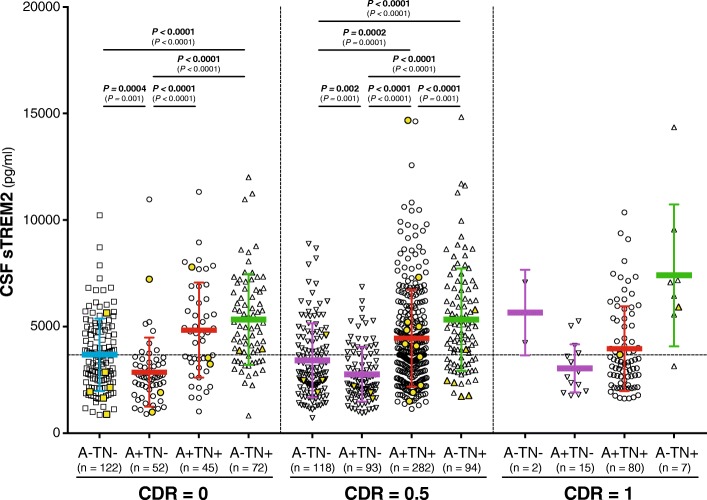
CSF sTREM2 in the A/T/N framework. Scatter plot depicting the levels of CSF sTREM2 for each of the four biomarker profiles, as defined by the A/T/N framework, coupled with clinical staging, as defined by CDR. The biomarkers groups T (tau pathology) and N (neurodegeneration) were merged in order to reduce the number of groups to compare. The CDR = 1 stage includes some biomarker profiles will low number of subjects, which precludes performing statistical analysis. They are still shown in the figure for sake of completeness. Each biomarker category is represented in a different colour: healthy controls are depicted in blue, Alzheimer’s *continuum* category in red, SNAP category in green, and purple depicts biomarker profiles not assigned in a specific category in the present study. The analysis reported in the main text was conducted excluding the *TREM2* rare variants carriers, the *P-*values are reported in bold, and the number of individuals (n) per group indicated. Including these carriers (depicted in yellow) rendered similar results (*P-*values reported between brackets). Solid bars represent the mean and the standard deviation (SD). *P*-values were assessed by a one-way ANCOVA adjusted for age, followed by Bonferroni corrected *post hoc* pairwise comparisons. Abbreviations: A: Aβ pathology biomarker status; AD: Alzheimer’s disease; CDR: clinical dementia rating; CSF: cerebrospinal fluid; N: neurodegeneration biomarker status; SNAP: suspected non-Alzheimer’s pathology; T: tau pathology biomarker status.

Within the CDR = 0 group (i.e. cognitively normal individuals), a one-way ANCOVA showed a significant difference between the four biomarker profiles after adjusting for the effect of age (F_3,286_ = 21.3, *P* < 0.0001, η_p_^2^ = 0.183). A Bonferroni *post hoc* test revealed that the A+/TN- profile had significant lower CSF sTREM2 compared to either other biomarker profile (Fig. 2). Only the A-/TN+ profile had significant higher CSF sTREM2 compared to the normal biomarkers profile (i.e. A-/TN-).

Within the CDR = 0.5 group (i.e. very mild dementia), there was also a significant difference between the four biomarker profiles (F_3,582_ = 40.7, *P* < 0.0001, η_p_^2^ = 0.173) and the A+/TN- profile had also the significantly lowest CSF sTREM2 compared to either other biomarker profile (Fig 2). Both the A+/TN+ and the A-/TN+ biomarker profiles had significant higher CSF sTREM2 compared to the A-/TN- profile (Fig. 2). The CDR = 1 group did not yield a sufficient number of subjects per A/T/N profile to allow for a group comparison.

We repeated the former analysis also including the individuals with *TREM2* rare variants (n = 43; demographics in Table S1, see Additional file 1) and this did not change our conclusions derived from the main analysis (Fig. 2).

We also repeated the same analysis classifying the participants based only on their Aβ pathology (A; CSF Aβ_1-42_) and tau pathology status (T; CSF P-tau_181P_), that is A/T classification, or based only on their Aβ pathology (A; CSF Aβ_1-42_) and neurodegeneration status (N; CSF T-tau), that is A/N classification. The results are shown in Figure S2 (see Additional file 1) and they are similar to those shown with the A/TN classification of the main text. Thus, we conclude that the pathological processes that are downstream of Aβ pathology, both tau pathology and neurodegeneration, are associated with increased CSF sTREM2.

### CSF sTREM2 changes across the Alzheimer’s *continuum*

**Fig. 3:**
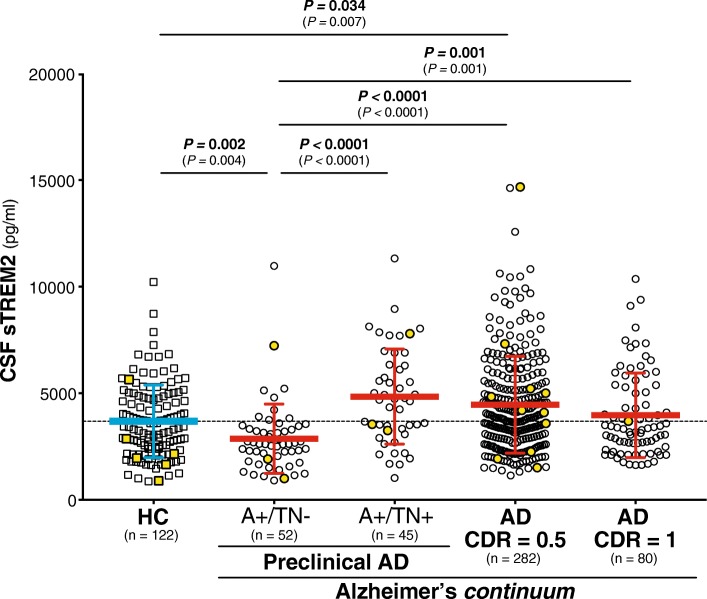
CSF sTREM2 across the Alzheimer’s *continuum*. Scatter plot showing the levels of CSF sTREM2 in healthy controls (depicted in blue) and the different stages of the Alzheimer’s *continuum* (depicted in red). The main analysis was conducted excluding the *TREM2* rare variants carriers, the *P-*values are reported in bold, and the number of individuals (n) per group indicated. Including these carriers (depicted in yellow) rendered similar results (*P-*values reported between brackets) *P*-values were assessed by a one-way ANCOVA adjusted for age, followed by Bonferroni corrected *post hoc* pairwise comparisons. Abbreviations: A: Aβ pathology biomarker status; AD: Alzheimer’s disease; CDR: clinical dementia rating; CSF: cerebrospinal fluid; N: neurodegeneration biomarker status; SNAP: suspected non-Alzheimer’s pathophysiology; T: tau pathology biomarker status.

Next we asked if CSF sTREM2 changes during the course of the disease as previously described in our study on late onset-AD [25]. We modeled the evolution of AD comparing the biomarker-defined groups (Table 1) that reflect the temporary course of late-onset AD, similar to what was proposed by the previous 2011 NIA-AA diagnostic criteria [32–34]. Thus, we compared the ‘healthy controls’ group (highlighted in blue in Table 1 and corresponding to the the ‘CDR = 0 A-/TN-’ group), with those belonging to the Alzheimer’s *continuum* (highlighted in red in Table 1), which included: ‘Preclinical AD A+/TN-’, ‘Preclinical AD A+/TN+’, ‘AD CDR = 0.5’ and ‘AD CDR = 1’. A one-way ANCOVA revealed that CSF sTREM2 was significantly different between groups after adjusting for the effect of age (F_4,575_ = 11.5, *P <* 0.0001, η_p_^2^ = 0.074). A *post hoc* analysis using the Bonferroni criterion for significance indicated that the average CSF sTREM2 was significantly higher in the ‘AD CDR = 0.5’ group than in the ‘healthy controls’ and ‘Preclinical AD A+TN-’ groups (*P =* 0.034 and *P <* 0.0001, respectively; Fig. 3). Similar results were obtained when the individuals carrying a *TREM2* rare variant were included (Fig. 3). Thus, these results replicate our and other groups previous findings of increased CSF sTREM2 in early symptomatic stages of late-onset AD in an independent sample [23–25, 29].

### CSF sTREM2 is associated with T-tau and P-tau but not Aβ_1-42_

**Fig 4.**
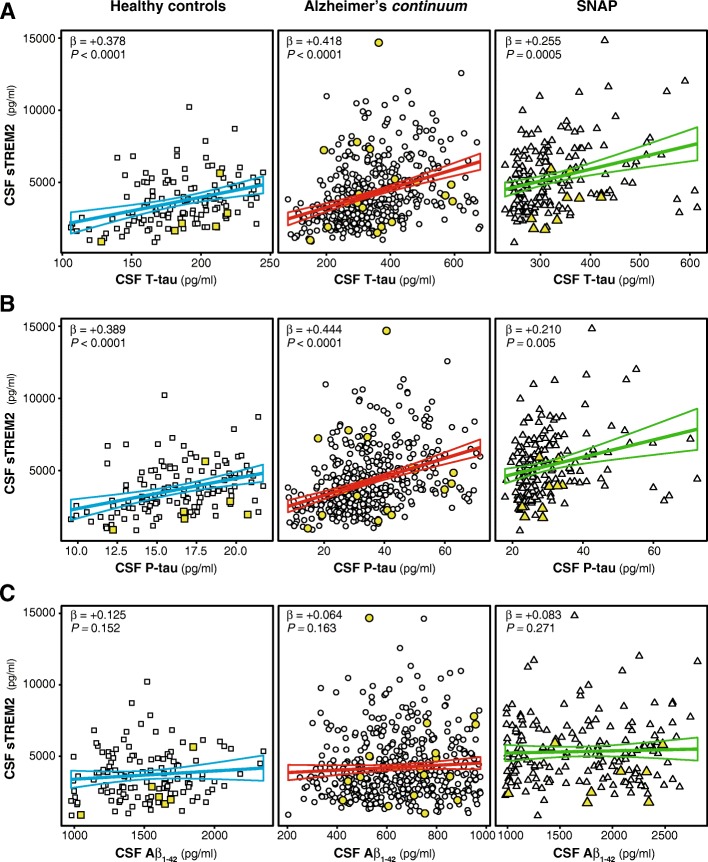
Association of CSF sTREM2 and AD core CSF biomarkers. Scatter plots representing the associations of CSF sTREM2 with each of the AD CSF core biomarkers: T-tau (**a**), P-tau_181P_ (**b**), and Aβ_1-42_ (**c**) in three different groups defined by the biomarker profile: healthy controls (blue), Alzheimer’s *continuum* (red) and SNAP groups (green). The solid lines indicate the regression line and the 95% confidence interval for each of the groups. The standardized regression coefficients (β) and the *P*-values are shown and were computed using a linear model adjusting for age, and are conducted excluding the outliers values. Including them, did not change the conclusions (see Additional file 1: Table S2). We also performed the analysis including the participants carrying a *TREM2* rare variant (depicted in yellow) and the results were similar (see Additional file 1: Table S3). Abbreviations: Aβ_1-42_: amyloid-beta 42; T-tau: total tau; P-tau: tau phosphorylated at Threonine 181; SNAP: suspected non-Alzheimer’s pathology.

Finally, we studied the associations of CSF sTREM2 with each of the CSF core biomarkers of AD, that is T-tau, P-tau_181P_ and Aβ_1-42_, in linear regression models adjusted for age. The associations were tested separately in three groups based on their biomarker profile (see Table 1): (1) healthy controls, (2) individuals of the Alzheimer’s *continuum* and (3) in the SNAP groups. Consistent with previous findings, CSF sTREM2 is associated with T-tau and P-tau_181P_ in the three groups studied (Fig. 4). In contrast, no significant associations were found between CSF sTREM2 and Aβ_1-42_ (Fig. 4). Including the CSF biomarkers outliers (see Additional file 1: Table S2), or including the individuals carrying a *TREM2* rare variants (see Additional file 1: Table S3), did not change our findings.

## Discussion

In the present study, we assessed the microglial-activity marker CSF sTREM2 within the early phases of AD. To this end, we applied the biomarker-based A/T/N classification in combination with clinical staging [27]. The use of this classification system enabled us to unravel the effect of Aβ pathology and its downstream processes (i.e. tau pathology and neurodegeneration) on the levels of CSF sTREM2. Interestingly, they are differentially associated with CSF sTREM2. While pure Aβ deposition (as defined here as low CSF Aβ_1-42_) is associated with decreased CSF sTREM2, tau pathology or neurodegeneration (as defined here as increased CSF P-tau_181P_ or CSF T-tau, respectively) are associated with an increase in CSF sTREM2. The higher CSF sTREM2 in the SNAP groups confirm that CSF sTREM2 rises with neurodegeneration without Aβ pathology.

Moreover, we show that the levels of CSF sTREM2 differ between *TREM2* variants and they are increased in the p.R47H *TREM2* variant compared to non-carriers, but decreased in the p.L211P variant and remained unchanged in the p.R62H variant, which is consistent with previous reports [24]. Increased levels of sTREM2 in the p.R47H variant were somewhat surprising; however, very little is known if that variant affects proteolytic processing of TREM2. In contrast, the increased levels of the p.H157Y variant is in line with our previous findings in cultured cells demonstrating that this variant increased shedding of TREM2 and therefore decreased TREM2 dependent phagocytosis [17]. A word of caution should be noted with the p.D87N variant, because the lower levels could be due, at least partially, to the lesser affinity of the antibody to this variant.

Among the strengths of this study are the large and well-characterized sample size and the use of a reliable assay to measure sTREM2. Yet, this study has some limitations. First, this is a cross-sectional study and the results need to be confirmed in a longitudinal setting. Second, we used the CSF biomarkers to classify the ADNI participants in the A/T/N classification. Although the role of CSF biomarkers in AD is well-established and a complete A/T/N characterization is possible only with CSF biomarkers, CSF T-tau may not necessarily reflect neurodegeneration but could result from physiological production of tau [46]. Here, we found CSF P-tau_181P_ and CSF T-tau to be highly correlated (only 5.4% of all the ADNI participants had a discrepant T and N biomarker group), and consequently we merged the “T” (tau pathology) and “N” (neurodegeneration) groups. Importantly, the TN- group had normal levels of both T-tau and P-tau_181P_, which reasonably ensure that other comorbidities that may cause neural injury (and hence microglial activation) are excluded. A plethora of other biofluid and neuroimaging markers (i.e. Aβ and tau PET, blood and CSF neurofilament light protein, anatomic MRI and FDG-PET) are in principle applicable for the implementation of the A/T/N classification, and future studies using these biomarkers are needed to confirm our results.

The A/T/N classification used herein is a descriptive biomarker-based classification that does not assume any temporal sequence of events in AD and is independent of the clinical stage of the disease. By applying this classification framework, we found an unexpected observation, namely a decrease of CSF sTREM2 in individuals with evidence of Aβ pathology but without signs of tau pathology or neurodegeneration. We did not observe this finding in previous studies, probably due to the low number of participants in the preclinical stage of late-onset AD and because we did not apply the A/T/N classification [25]. Noteworthy, we previously observed in ADAD that CSF sTREM2 was lower in ADAD mutation carriers than in non-carriers at very early stages (EYO < -15; CSF T-tau becomes significantly increased in mutation carriers at EYO = -15), yet statistically non-significant [26]. In contrast, we show here that CSF sTREM2 increases as soon there are signs of we show here that CSF sTREM2 increases, both in the context of AD (that is with co-occurrence of Aβ pathology) or in the SNAP patients (where there is no Aβ underlying pathology). Consistent with these findings, CSF sTREM2 is distinctly associated with CSF T-tau and CSF P-tau_181P_, but not with CSF Aβ_1-42_. A word of caution is needed in the SNAP category, given that this is an heterogenous group that most likely exhibits a non-AD related neurodegeneration. Well-powered future studies should address how CSF sTREM2 changes in neurodegenerative diseases different from AD.

The flexibility of this new classification framework also enabled us to model the natural history of AD and confirm in the ADNI study our previous findings in participants of several European memory clinics [25] who were classified using the 2011 NIA-AA criteria [32–34, 36]. Herein, we demonstrate that, after the initial decrease of CSF sTREM2 in the ‘Preclinical AD A+/TN-’ group, CSF sTREM2 rise is the ‘Preclinical AD A+/TN+’group and in the early symptomatic stage (CDR = 0.5) of AD, albeit only statistically significant in the latter group. These findings therefore replicate our [25, 26] and other groups [23, 24, 29] previous findings in which an increase in CSF sTREM2 in early symptomatic AD was observed.

The mechanism underlying the dynamic changes of CSF sTREM2 throughout the course of the disease still need to be investigated. Several studies have consistently demonstrated that microglia upregulate TREM2 expression in AD mouse models and in human AD brains [3, 47, 48]. Moreover, detailed transcriptomics studies that investigated microglia in mouse models of AD and neurodegeneration showed that TREM2 is upregulated in the disease-associated microglia (DAM) [1, 49–53]. This is consistent with the finding of increased CSF sTREM2 in stages downstream of Aβ accumulation, that is when tau pathology and/or neurodegeneration occur and microglia may adopt their disease associated molecular signature. We were surprised, however, by the observation of an initial CSF sTREM2 decrease in the only Aβ-pathology stage, which corresponds to the earliest stage of the disease. The possible mechanisms behind this observation are still elusive. However, it has been described that microglia are activated in two steps with an initial TREM2-independent process followed by a TREM2-dependent process [49]. The CSF sTREM2 increase observed following the initially low levels may reflect the second step of activation. An alternative explanation would be that microglia initially forms a barrier around plaques [54, 55] and the sTREM2 released by microglia is retained within the plaque, until the barrier fails, and subsequent neural injury starts. Finally, it could also be argued that subjects with low TREM2-function (and hence lower CSF sTREM2 levels) are more prone to experience an accelerated early amyloidogenesis (Parhizkar *et al.* Nat. Neursci *in press*) and are therefore overrepresented in the Preclinical AD A+TN- group. Nevertheless, we are cognizant of the fact that this is an observational study and the findings reported herein do not elucidate precise mechanisms. Further work with longitudinal data is needed to address whether the stage-dependent changes in CSF sTREM2 predict a better or worse clinical outcome.

In conclusion, the present study represents the first attempt to study CSF sTREM2 based on the A/T/N classification framework. We demonstrate in the ADNI cohort that the increase in CSF sTREM2 which occurs in early stages parallels the increase in biomarkers of tau pathology and neurodegeneration. In contrast, Aβ deposition in the absence of tau deposition and neurodegeneration is associated with lower CSF sTREM2.

## Additional file

Additional file 1: CSF sTREM2 measurement. **Figure S1.** Effects of rare *TREM2* variants on antibody affinities. **Figure S2.** CSF sTREM2 in the A/T and in the A/N classifications. **Table S1.** Demographic and clinical characteristics of the entire sample. **Table S2.** Associations of CSF sTREM2 with AD CSF core biomarkers including the biomarkers outliers. **Table S3.** Associations of CSF sTREM2 with AD CSF core biomarkers including subjects carrying a TREM2 rare variant. (DOCX 1992 kb)

## Abbreviations

Aβ: Amyloid β-peptide
AD: Alzheimer’s disease;
ADAD: Autosomal-dominant Alzheimer’s disease
CDR: Clinical dementia rating score
CNS: Central nervous system
CSF: Cerebrospinal fluid
EYO: Estimated years from symptom onset
SNAP: Suspected non-Alzheimer’s pathology
sTREM2: Soluble triggering receptor expressed on myeloid cells 2
TREM2: Triggering receptor expressed on myeloid cells 2
