# Supplementary material for: Early increase of CSF sTREM2 in Alzheimer’s disease is associated with tau related-neurodegeneration but not with amyloid-β pathology
Source: Mol Neurodegener. 2019 Jan 10;14:1. doi: 10.1186/s13024-018-0301-5 (PMC6327425; doi:10.1186/s13024-018-0301-5)
Supplement: Supplementary file 1 — CSF sTREM2 measurement. Figure S1. Effects of rare TREM2 variants on antibody affinities. Figure S2. CSF sTREM2 in the A/T and in the A/N classifications. Table S1. Demographic and clinical characteristics of the entire sample. Table S2. Associations of CSF sTREM2 with AD CSF core biomarkers including the biomarkers outliers. Table S3. Associations of CSF sTREM2 with AD CSF core biomarkers including subjects carrying a TREM2 rare variant. (DOCX 1992 kb) [file 13024_2018_301_MOESM1_ESM.docx]

**Supplementary methods**

**CSF sTREM2 measurement**

CSF sTREM2 measurements in ADNI were done with an MSD platform-based assay previously reported [1–3] with minor changes. Briefly, the assay consists of a Streptavidin-coated 96-well plate (MSD Streptavidin Gold Plates, cat. no. L15SA); a biotinylated polyclonal goat IgG anti-human TREM2 antibody (R&D Systems, cat. no. BAF1828; 0.25 µg/mL, 25 µL/well) as capture antibody, which is raised against aminoacids 19-174 of human TREM2; a monoclonal mouse IgG anti-human TREM2 antibody (Santa Cruz Biotechnology, B-3, cat. no. sc373828; 1 µg/mL, 50 µL/well) as a detection antibody, which is raised against aminoacids 1-160 of human TREM2; and a SULFO-TAG-labeled goat polyclonal anti-mouse IgG secondary antibody (MSD, cat. no. R32AC; 0.5 µg/mL, 25 µL/well). All antibodies were diluted in 1% BSA and 0.05% Tween 20 in PBS (pH = 7.4) buffer. Recombinant human TREM2 protein (Hölzel Diagnostika, cat. no. 11084-H08H), corresponding to the extracellular domain of human TREM2 (aminoacids 19-174) was used as a standard (62.5 to 8000 pg/mL). Streptavidin-coated 96-well plates were blocked overnight at 4°C in blocking buffer [3% bovine serum albumin (BSA) and 0.05% Tween 20 in PBS (pH = 7.4); 300 μL/well]. The plates were next incubated with the capture antibody for 1.5 hour at room temperature (RT). They were subsequently washed four times with washing buffer (200 μL/well; 0.05% Tween 20 in PBS). Thereafter, the recombinant human TREM2 protein (standard curve), the blanks, and the CSF samples and the internal standards IS (duplicates; dilution factor: 4) were diluted in assay buffer [0.25% BSA and 0.05% Tween 20 in PBS (pH = 7.4)] supplemented with protease inhibitors (Sigma; cat. no. P8340) and incubated (50 μL/well) for 2 hours at RT. This dilution was previously selected because it showed the best recovery and linearity performance [1]. Plates were again washed four times with washing buffer before incubation for 1 hour at RT with detection antibody. After four additional washing steps, plates were incubated with SULFO-tag conjugated secondary antibody for 1 hour in the dark at RT. Last, plates were washed four times with wash buffer followed by two washing steps in PBS. The electrochemical signal was developed by adding 150 μL/well MSD Read buffer T (cat. no. R-92TC) and the light emission measured using the MESO QuickPlex SQ 120. Raw values are provided as pg/mL.

All CSF samples were distributed randomly across plates and measured in duplicate between the 27/11/2017 and 06/12/2017. The mean intraplate CV was 3.1% and all duplicate measures had a CV < 15%. Four CSF internal standards (IS) were loaded in all plates. All IS used in this study consisted of pooled CSFs from diagnostic clinical routine leftovers from the Ludwig-Maximilians-Universität München (LMU) department of Neurology (Munich, Germany). All patients gave their written consent and the study was approved by the local IRB. The interplate CV for each of the IS were 11.4%, 12.2%, 10.5% and 7.1%. We corrected the raw measurements based on values of the four IS that were loaded on all plates. The concentration of each IS in an individual plate (plate x) was expressed as a percentage of the mean concentration across all plates, as follows:

**a1** (%) in plate x = [(concentration of IS1 in plate x) / (mean concentration of IS1 in all plates)] ×100

**a2** (%) in plate x = [(concentration of IS2 in plate x) / (mean concentration of IS2 in all plates)] ×100

**a3** (%) in plate x = [(concentration of IS3 in plate x) / (mean concentration of IS3 in all plates)] ×100

**a4** (%) in plate x = [(concentration of IS4 in plate x) / (mean concentration of IS4 in all plates)] ×100

The mean of the percentages (**Ax**) for all the IS (**a1, a2, a3, a4**) in plate x was calculated and the following correction factor was computed for each individual plate:

**Correction factor for plate x** = 100 / **Ax**

The raw values were multiplied by the correction factor of the corresponding plate; the corrected values were used and are publicly available in the ADNI database as variables ‘MSD_sTREM2CORRECTED’.

**Supplementary results**

**Figure S1.**

**Fig S1. Effects of rare *TREM2* variants on antibody affinities.**

HEK293T cells were transfected with N-terminal HA-tagged *TREM2* constructs bearing different *TREM2* rare variants (p.R47H, p.L211P, p.R62H, p.D87N and p.H157Y). The HA-tagged sTREM2 released in the conditioned media was quantified by two ELISAs: (1) the same MSD platform-based sTREM2 ELISA used for the quantification of the human CSF samples, which includes the Santa Cruz Biotechnology B-3 monoclonal mouse IgG anti-human TREM2 antibody (cat. no. sc-373828; yellow bars); (2) a modified MSD platform-based ELISA using as a detection antibody the 3F10 monoclonal rat IgG anti-HA antibody (purple bars). Each bar depicts the electrochemiluminescence (ECL) response acquired. The response of the sTREM2 ELISA (B-3 detection antibody) for each of the TREM2 rare variants is expressed normalized to the respective HA-tagged ELISA control. The bars chart and error bars show the mean and standard deviation of four technical replicates. Differences on the antibody affinities for each of the *TREM2* variants were compared with a Mann-Whitney test.

Abbreviations: *wt*, wild-type.

**Figure S2**

**Fig S2. CSF sTREM2 in the A/T and in the A/N classifications**

(A) A/T classification: ADNI participants were classified based on their Aβ pathology (A; CSF Aβ_1−42_) and tau pathology status (T; CSF P-tau). (B) A/N classification: ADNI participants were classified based on their Aβ pathology (A; CSF Aβ_1−42_) and neurodegeneration status (N; CSF T-tau).

Solid bars represent the mean and the standard deviation (SD). *P*-values were assessed by a one-way analysis of covariance adjusted for age, followed by Bonferroni corrected *post hoc* pairwise comparisons. The *P-*values of the analysis conducted excluding the *TREM2* rare variants are reported in bold. The *P-*values including the *TREM2* rare variants are reported between brackets. The CDR = 1 stage includes some biomarker profiles will low number of subjects that precluded performing statistical analysis.

Abbreviations: A: Aβ pathology biomarker status; AD: Alzheimer’s disease; CDR: clinical dementia rating; CSF: cerebrospinal fluid; N: neurodegeneration biomarker status; SNAP: suspected non-Alzheimer’s pathology; T: tau pathology biomarker status.

**Supplementary Tables**

**Table S1. Demographic and clinical characteristics of the entire sample**

|  | **CDR = 0**  (n = 307) | | | | **CDR = 0.5**  (n = 613) | | | | **CDR = 1**  (n = 107) | | | |
| --- | --- | --- | --- | --- | --- | --- | --- | --- | --- | --- | --- | --- |
|  | **A-/TN-**  (n = 128) | **A+/TN-**  (n = 57) | **A+/TN+**  (n = 48) | **A-/TN+**  (n = 74) | **A-/TN-**  (n = 122) | **A+/TN-**  (n = 97) | **A+/TN+**  (n = 294) | **A-/TN+**  (n = 100) | **A-/TN-**  (n = 2) | **A+/TN-**  (n = 15) | **A+/TN+**  (n = 82) | **A-/TN+**  (n = 8) |
| Age, y | 72.5 (5.40) | 73.2 (5.91) | 76.5 (5.37) | 74.6 (6.6) | 69.9 (7.61) | 72.7 (7.67) | 73.4 (7.06) | 73.3 (8.12) | 89.2 (1.63) | 76.3 (6.05) | 73.9 (9.26) | 79.9 (7.43) |
| Female, n (%) | 62 (48.4) | 27 (47.4) | 25 (52.1) | 41 (55.4) | 56 (45.9) | 21 (21.7) | 125 (42.5) | 46 (46.0) | 0 (0) | 5 (33.3) | 41 (50.0) | 2 (25.0) |
| *APOE* ε4 carriers, n (%) | 18 (14.1) | 22 (38.6) | 28 (58.3) | 16 (21.6) | 25 (20.5) | 48 (49.5) | 224 (76.2) | 33 (33.0) | 0 (0) | 8 (53.3) | 61 (74.4) | 1 (12.5) |
| *TREM2* rare variants*,* n (%) | 6 (4.69) | 4 (7.02) | 3 (6.25) | 2 (2.70) | 4 (3.28) | 4 (4.12) | 12 (4.08) | 6 (6.00) | 0 (0) | 0 (0) | 1 (1.22) | 1 (12.5) |
| Education, y | 16.3 (2.79) | 16.4 (2.73) | 16.5 (2.47) | 16.4 (2.56) | 16.1 (2.68) | 16.1 (2.98) | 15.9 (2.87) | 15.9 (2.62) | 17.0 (0.0) | 16.2 (2.51) | 15.1 (2.77) | 14.9 (1.96) |
| **CSF biomarkers, pg/ml*** |  |  |  |  |  |  |  |  |  |  |  |  |
| T-tau | 185 (32.2) | 165 (42.4) | 329 (77.5) | 324 (72.2) | 184 (37.7) | 171 (40.2) | 383 (135) | 337 (108) | 234 (6.08) | 188 (38.8) | 394 (137) | 468 (213) |
| P-tau_181P_ | 16.3 (2.89) | 15.3 (4.17) | 33.0 (8.93) | 29.0 (7.36) | 15.8 (3.28) | 15.8 (4.19) | 39.2 (14.6) | 31.4 (13.3) | 19.3 (1.20) | 17.4 (3.53) | 39.3 (15.1) | 41.3 (21.9) |
| Aβ_1-42_ | 1460 (224) | 719 (196) | 712 (173) | 1542 (233) | 1432 (246) | 640 (198) | 637 (167) | 1437 (291) | 1330 (14.9) | 538 (185) | 574 (159) | 1480 (269) |
| sTREM2 | 3684 (1702) | 2784 (1648) | 4840 (2230) | 5339 (2130) | 3417 (1738) | 2740 (1295) | 4513 (2441) | 5330 (2398) | 5660 (2008) | 3051 (1128) | 3922 (2010) | 7406 (3331) |

Data are expressed as mean and standard deviation (SD) or number (n) and percentage (%), as appropriate.

*The CSF core biomarkers measurements were performed using the electrochemiluminiscence immunoassays Elecsys Total-tau CSF, phosphor-tau(181P) CSF and β-amyloid(1-42) CSF, which have an upper technical limit of 1300 pg/ml (T-tau), 120pg/ml (P-tau_181P_) or 1700 pg/ml (Aβ_1-42_). The values above these limits were truncated to the respective upper technical limit.

Abbreviations: A: Aβ pathology biomarker status; Aβ_1-42_: amyloid-β 42; APOE, apolipoprotein E; CDR: clinical dementia rating; CSF: cerebrospinal fluid; N: neurodegeneration biomarker status; P-tau_181P_, tau phosphorylated at threonine 181; T: tau pathology biomarker status; T-tau: total tau; y, years.

**Table S2. Associations of CSF sTREM2 with AD CSF core biomarkers including the biomarkers outliers**

|  | **Healthy controls**  (n = 122) | **Alzheimer’s *continuum***  (n = 461) | **SNAP**  (n = 173) |
| --- | --- | --- | --- |
| T-tau | β = +0.378  *P* < 0.0001 | β = +0.401  *P* < 0.0001 | β = +0.333  *P* < 0.0001 |
| P-tau_181P_ | β = +0.389  *P* < 0.0001 | β = +0.401  *P* < 0.0001 | β = +0.293  *P* < 0.0001 |
| Aβ_1-42_ | β = +0.125  *P* = 0.152 | β = +0.057  *P* = 0.211 | β = +0.149  *P* = 0.043 |

The standardized regression coefficients (β) and the *P*-values are shown and were computed using a linear model adjusting for age. The Aβ_1-42_ values used for the associations test are those based on an extrapolation curve since the upper technical limit is 1700pg/ml.

Abbreviations: Aβ_1-42_: amyloid-beta 42; T-tau: total tau; P-tau_181P_: tau phosphorylated at Threonine 181; SNAP: suspected non-Alzheimer’s pathology.

**Table S3. Associations of CSF sTREM2 with AD CSF core biomarkers including subjects carrying a *TREM2* rare variant**

|  | **Healthy controls**  (n = 128) | **Alzheimer’s *continuum***  (n = 477) | **SNAP**  (n = 182) |
| --- | --- | --- | --- |
| T-tau | β = +0.383  *P* < 0.0001 | β = +0.408  *P* < 0.0001 | β = +0.247  *P* = 0.0006 |
| P-tau_181P_ | β = +0.371  *P* < 0.0001 | β = +0.434  *P* < 0.0001 | β = +0.213  *P* = 0.003 |
| Aβ_1-42_ | β = +0.145  *P* = 0.090 | β = +0.068  *P* = 0.129 | β = +0.061  *P* = 0.411 |

The standardized regression coefficients (β) and the *P*-values are shown and were computed using a linear model adjusting for age. The Aβ_1-42_ values used for the associations test are those based on an extrapolation curve since the upper technical limit is 1700pg/ml.

Abbreviations: Aβ_1-42_: amyloid-beta 42; T-tau: total tau; P-tau_181P_: tau phosphorylated at Threonine 181; SNAP: suspected non-Alzheimer’s pathology.

**Supplementary References**

1. Kleinberger G, Yamanishi Y, Suárez-Calvet M, Czirr E, Lohmann E, Cuyvers E, et al. TREM2 mutations implicated in neurodegeneration impair cell surface transport and phagocytosis. Sci Transl Med. 2014;6:243ra86.

2. Suárez‐Calvet M, Kleinberger G, Araque Caballero MÁ, Brendel M, Rominger A, Alcolea D, et al. sTREM2 cerebrospinal fluid levels are a potential biomarker for microglia activity in early‐stage Alzheimer’s disease and associate with neuronal injury markers. EMBO Mol Med. 2016;8:466–76.

3. Suárez-Calvet M, Araque Caballero MÁ, Kleinberger G, Bateman RJ, Fagan AM, Morris JC, et al. Early changes in CSF sTREM2 in dominantly inherited Alzheimer’s disease occur after amyloid deposition and neuronal injury. Sci Transl Med. 2016;8:369ra178.
